# The risk of lymph node metastasis in gastric cancer conforming to indications of endoscopic resection and pylorus-preserving gastrectomy: a single-center retrospective study

**DOI:** 10.1186/s12885-021-09008-8

**Published:** 2021-11-27

**Authors:** Wu Yanzhang, Li Guanghua, Zhou Zhihao, Wang Zhixiong, Wang Zhao

**Affiliations:** Department of Gastrointestinal Surgery, First Affiliated Hospital of Sun Yat-sen University, Zhongshan 2nd street, No. 58, Guangzhou, 510080 Guangdong China

**Keywords:** Lymph node metastasis, Lymphatic invasion, Skip metastasis, Early gastric cancer, Predictive model

## Abstract

**Background:**

Lymph node metastasis (LNM) status is an important prognostic factor that strongly influences the treatment decision of early gastric cancer (EGC). This study aimed to evaluate the pattern and clinical significance of LNM in EGC.

**Methods:**

A total of 354 patients with carcinoma in situ (*n* = 42), EGC (*n* = 312) who underwent radical gastrectomy were enrolled. Their clinicopathological features, pathological reports, and prognostic data were collected and analyzed.

**Results:**

The incidence of LNM in all patients was 18.36% (65/354). The rates of D1 and D2 station metastases were 12.10% (43/354) and 6.21% (22/354), respectively. The rates of LNM in absolute indication of endoscopic resection and expanded indication were 3.27% (2/61) and 28.55% (4/14), respectively. Skip LNM was observed in 3.67% (13/354) of patients. For those with middle-third tumor, the metastasis rate of the No. 5 lymph node was 3.05% (5/164). The independent risk factors for LNM were tumors measuring > 30 mm, poorly differentiated tumors, and lymphovascular invasion (all *P* < 0.05; area under the curve, 0.783). The 5-year disease-free survival rates of patients with and without LNM were 96.26 and 79.17%, respectively (*P* = 0.011). Tumors measuring > 20 mm and LNM were independent predictive factors for poor survival outcome in all patients.

**Conclusions:**

Patients with EGC conforming to expanded indications have a relatively high risk of LNM and may not be suitable for endoscopic submucosal dissection. Pylorus-preserving gastrectomy for patients with middle-third EGC remains controversial due to the high metastasis rate of the No. 5 lymph node.

## Background

Gastric cancer (GC) is the fifth most common cancer type and the third leading cause of cancer-related mortality worldwide [1]. About 75% of cases appear in Asia, particularly in China, Korea, and Japan. China accounted for 50% of the new cases [2]. Over the past several decades, these Eastern Asian countries have made great efforts to prolong the survival time and improve the quality of life of patients with GC. One of the great achievements is the improvement of screening strategies for early GC (EGC) detection. It has been reported that the detection rate of EGC increased to 61% in Korea [3].

EGC has been defined as invasive gastric cancer confined to the mucosa or submucosa layer of the stomach, regardless of lymph node metastasis (LNM) [4]. Compared to advanced GC, EGC has a high 5-year survival rate, up to 99%. D2 lymphadenectomy with gastrectomy has been the standard surgical procedure for advanced GC. The treatment decision for EGC seems to be complicated, diversified, and controversial compared with that of advanced GC. According to the 2018 Japanese GC treatment guidelines [5], endoscopic resection (ER) has been recommended as an alternative curative treatment for patients with EGC with indications. In addition to ER treatment, some modified surgical procedures, such as pylorus-preserving gastrectomy (PPG), and segmental gastrectomy, can be considered for EGC with a low risk of LNM and are not suitable for ER to improve the quality of life.

LNM status is an important prognostic factor of EGC [6–8]. According to the 2018 Japanese GC treatment guidelines [5], the choice of ER for EGC treatment is mainly dependent on the risk of LNM. LNM in patients with EGC within absolute indications for endoscopic mucosal resection (EMR) or endoscopic submucosal dissection (ESD) has been hypothesized to be negligible (< 1%). Currently, ESD is widely used as a standard method for EGC in Japan, and its indications are expanded. However, several problems remain. First, although most evidence suggests that the risk of LNM in patients with absolute indications is negligible, the results between these studies were inconsistent [5]. Second, the expanded indications for ESD remain controversial. Third, the management of cases with noncurative resection after ER is controversial.

Furthermore, there have been relatively few studies on the positive rate of each lymph node station and skip metastasis in EGC [9–13]. The definition of skip metastasis in GC cases refers to the presence of extraperigastric LNM without perigastric lymph node involvement [10]. In the present study, we aimed to elucidate the precise distribution of LNM by analyzing the metastasis status of each lymph node station in patients with EGC who underwent D2 lymphadenectomy with gastrectomy and to explore the clinical significance of LNM pattern and skip metastasis in making treatment decisions for EGC.

## Methods

### Patient cohort and data collection

The clinicopathological data of patients (*n* = 2245) who underwent D2 lymphadenectomy gastrectomy at the First Affiliated Hospital of Sun Yat-Sen University (January 2010 to December 2018) were retrospectively analyzed. All surgical procedures involved resection of at least two-thirds of the stomach with D2 lymph node dissection and were performed according to the guidelines of the Japanese Gastric Cancer Association [5]. No patients agreed to undergo ESD/EMR before the surgery. All clinicopathologic data, including age, sex, tumor location, tumor size, histological classification, lymphovascular invasion (LVI), depth of tumor invasion, and LNM, were collected from hospital and pathological records. Staging was performed according to the corresponding seventh edition of the AJCC Staging Manual [14]. Well- and moderately differentiated tubular adenocarcinomas and papillary adenocarcinomas were grouped together as “differentiated lesions.” Poorly differentiated adenocarcinomas and signet-ring cell carcinomas were classified as “undifferentiated histological types.” Lesions with ulceration or scarring from previous ulceration (converging folds or deformity of the muscularis propria, or fibrosis in the submucosal or deeper layer) within them were regarded as “ulcerated lesions” [15]. This study was approved by the Ethics Committee of the First Affiliated Hospital of Sun Yat-sen University and conducted in accordance with the principles of the Declaration of Helsinki. The need for informed consent for participation and for approval of all patients was waived owing to the retrospective nature of the study and anonymized data.

### Inclusion and exclusion criteria

**Fig. 1:**
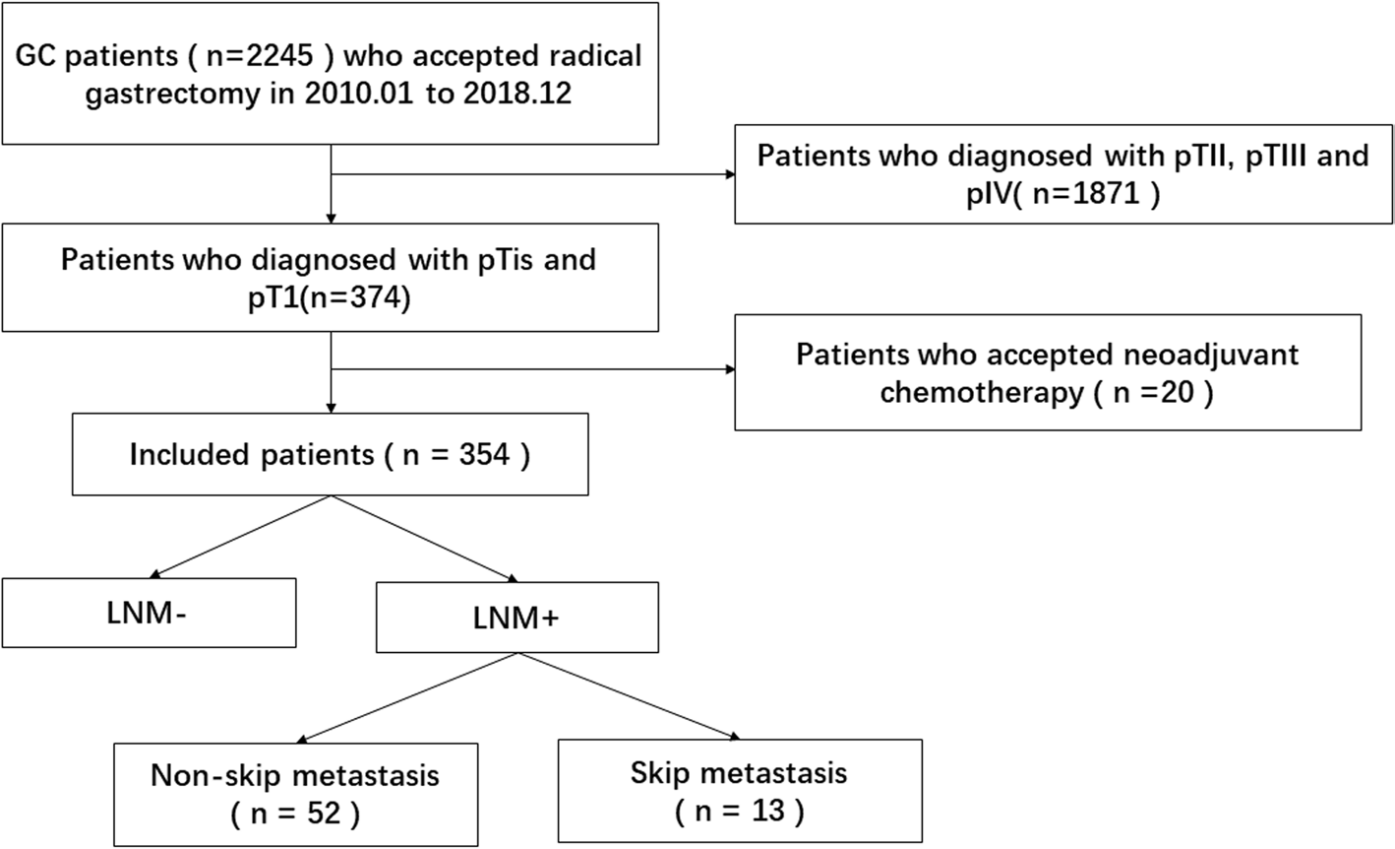
Inclusion criteria for study participants. LNM-, absence of lymph node metastasis; LNM+, presence of lymph node metastasis

The inclusion criteria were as follows: (1) the depth of invasion was diagnosed as carcinoma in situ (Tis), mucosa (T1a), or submucosa (T1b); and (2) absence of distant metastasis. Patients were excluded when they had (1) received neoadjuvant therapy or (2) incomplete clinicopathologic information (Fig. 1).

In total, 354 cases histologically proven to be Tis, T1a or T1b following the inclusion and exclusion criteria were enrolled (Fig. 1). All patients were divided into the LNM+ group (*n* = 65, LNM+: presence of LNM) or LNM– group (*n* = 289, LNM-: absence of LNM). To analyze the LNM rate for the patients selected by the indications of ESD/EMR, all patients were also divided into four different groups according to the absolute and expanded indications of ESD/EMR. For submucosal invasive (T1b) EGC, the LNM status was analyzed according to two conditions (≤2 cm, differentiated type; ≤ 2 cm, undifferentiated type).

### Follow-up examinations

All patients included in this study were regularly followed up using a standardized protocol. Follow-up assessment included abdominal ultrasonography, computed tomography (CT) imaging (of the chest, abdomen and pelvis), and tumor marker tests (including cancer antigen [CA]-19–9, carcinoembryonic antigen [CEA], CA125, squamous cell carcinoma) at each visit.

Regarding the overall survival analysis, deaths due to any reasons were recorded as events. Regarding the disease-free survival analysis, deaths due to cancer were recorded as events, which were defined as postoperative recurrences at any site or cancer-related death. Deaths secondary to other causes, such as another disease or accident, were censored. Patients with unknown causes of death and their postoperative complications were excluded from the prognostic analysis.

### Statistical analysis

All statistical analyses were conducted using the SPSS 24.0 statistical package (IBM Inc., Armonk, NY, USA) and R (https://www.r-project.org/; R Foundation for Statistical Computing, Vienna, Austria). Continuous variables are presented as the mean and standard deviation, and an analysis of variance test was used to compare continuous variables. For categorical variables, Pearson’s chi-square or Fisher’s exact test was used to compare the differences between the patient groups. Univariate and multivariate logistic regression models, in which all covariates were adjusted simultaneously, were used to determine independent risk factors for LNM. Kaplan–Meier curves were plotted to evaluate the survival outcomes in patients, and comparisons of prognostic differences between the patient groups were performed using the log-rank test. Independent prognostic factors were identified by multivariate analysis using the Cox proportional-hazard model with a stepwise selection procedure. Hazard ratios (HRs) and 95% confidence intervals (CIs) were calculated to quantify the relationship between the survival outcome and each clinicopathologic factor. Statistical significance was accepted at a *P* value < 0.05.

## Results

### Clinicopathological characteristics of patients according to LNM

The data of 2245 patients with GC who underwent radical gastrectomy with lymphadenectomy at the First Affiliated Hospital of Sun Yat-Sen University between January 1, 2010 and December 31, 2018 were reviewed retrospectively. In total, 354 cases histologically proven to be EGC or carcinoma in situ according to the inclusion and exclusion criteria were enrolled for the next analysis. As shown in Table 1, the current study consisted of 224 male (63.27%) and 130 female patients (36.72%), with a median age of 57.50 ± 11.399 years (range, 24–85 years). Among these 354 patients, 35 (9.89%), 165 (46.61%), and 175 (49.43%) patients had tumors located in the upper third, middle third, and lower third of the stomach, respectively. The mean length and short diameter of the tumor were 2.254 ± 1.344 cm and 1.808 ± 1.184 cm, respectively. Postoperative pathology indicated LVI in 16 cases (4.52%) and poorly differentiated tumors in 185 cases (52.26%). The average number of lymph node dissections was 37.640 ± 23.203.

**Table 1:** Clinicopathological characteristics in the LNM- (*n* = 289) and LNM+ groups (*n* = 65)

Factor	LNM- (*n* = 289)	LNM+ (*n* = 65)	LNM%	Relative risk (CI 95%)	*P*-value
Age (years)	56.74 ± 11.084	54.80 ± 12.677			0.301
< 40	16	10	38.46%	2.297 (1.333–3.947)	0.006
≥ 40	273	55	16.77%		
Sex					0.373
Male	186	38	16.96%		
Female	103	27	20.77%		
BMI (kg/m^2^)	21.08 ± 5.914	21.24 ± 5.782			0.874
Size (cm)					
Length-diameter	2.091 ± 1.198	2.930 ± 1.689			0.009
Short-diameter	1.713 ± 1.126	2.196 ± 1.342			0.068
< 2 cm	152	26	14.61%	1.712 (0.924–3.174)	0.076
≥ 2 cm	137	39	22.16%		
< 3 cm	236	39	14.18%	2.496 (1.532–4.065)	< 0.001
≥ 3 cm	53	26	32.91%		
Tumor marker					
CEA (U/mL)	7.032 ± 81.39	4.394 ± 9.618			0.631
CA125 (U/mL)	10.31 ± 9.527	9.995 ± 5.774			0.566
CA199 (U/mL)	13.689 ± 59.54	17.703 ± 45.017			0.526
Location					
Upper	27	6	18.18%		0.765
Middle	136	28	17.07%		
Lower	142	31	17.92%		
Depth of invasion					
Tis	41	1	2.38%		< 0.001
T1a	152	25	14.12%		
T1b	98	37	27.41%		
Differentiation					
Well/Moderate	155	14	8.28%		< 0.001
Poorly	134	51	27.57%	3.328 (1.914–5.787)	
Ulcer finding					
Absent	133	36	21.30%		0.172
Present	156	29	15.68%		
Number of lymph nodes	36.97 ± 24.157	40.63 ± 18.23			0.864
LVI					
Absent	283	55	16.27%		< 0.001
Present	6	10	62.5%	3.751 (2.422–5.809)	
Recurrence	7	2			
OS rate^a^	94.54%	80.77%			0.021
DFS rate^a^	93.64%	79.17%			0.011

*BMI* body mass index, *LNM* lymph node metastasis, *LVI* lymphovascular invasion, *LNM-* absence of lymph node metastasis, *LNM+* presence of lymph node metastasis, *CI* confidence interval, *CEA* carcinoembryonic antigen, *CA125* cancer antigen 125, *CA199* cancer antigen 199, *OS* overall survival, *DFS* disease-free survival

^a^The 5-year survival rate refers to the survival status of patients treated with surgery between January 2010 and March 2015. The OS rates were as follows: LNM-, 104/107 (94.54%); LNM+, 20/24 (80.77%). The DFS rates were as follows: LNM-, 103/107 (96.26%); LNM+, 19/24 (79.17%)

There were 61.86% (219/354) patients with intra-mucosal invasion (including Tis and T1a) and 38.14% (135/354) patients with submucosa (T1b) invasion. The percentages of LNM positivity was 27.41% (37/135) in the submucosa group (*P* < 0.001). Meanwhile, the percentages of LNM positivity were 2.38% (1/42) in the Tis groups and 14.12% (25/177) in the T1a groups. There was no significant difference in the mean age of patients between the two groups, but there was a significant difference between those aged < 40 and ≥ 40 years (*P* = 0.006), suggesting that younger patients have a higher risk of presenting LNM (risk ratio [RR] = 2.297; 95% CI, 1.333–3.947). Tumor sizes were significantly larger for LNM+ than for LNM− cases (*P* = 0.009). Compared with those with LNM−, tumor invasion was deeper (*P* < 0.001; RR = 2.256; 95% CI, 1.447–3.518) and showed poor differentiation (P < 0.001; RR = 3.328; 95% CI, 1.914–5.787) in those with LNM+. However, the distribution of other variables including sex, body mass index, tumor maker, and tumor location were similar between the LNM− and LNM+ groups.

### Metastasis status of different lymph node groups in patients

In this study, the incidence of LNM in these patients was 18.36% (65/354). To further elucidate the role of LNM in EGC, we analyzed the positive rate (Table 2) and location distribution (Table 3) of LNM for each lymph node station. As shown in Table 2, the positive rates of No. 3, 4, and 6 lymph nodes were 4.80, 3.67, and 3.95%, respectively, regardless of the tumor location. For tumors located in the upper-third of the stomach with LNM (*n* = 6), the No. 2 and 3 lymph nodes had high positive rates of LNM (Table 3). For tumors in the middle-third of the stomach (*n* = 28), No. 3, 4, 5, and 6 lymph nodes had the highest positive rates of LNM. For tumors in the lower third of the stomach, No. 3 and 6 lymph node stations had the highest metastasis rates.

**Table 2:** Positive rate of each lymph node station in all patients (*n* = 354)

Station	Case	Positive rate	Station	Case	Positive rate
No. 1	9	2.54%	No. 7	10	2.82%
No. 2	2	0.56%	No. 8	6	1.69%
			No. 8a	5	1.41%
			No. 8p	1	0.28%
No. 3	17	4.80%	No. 9	2	0.56%
No. 4	13	3.67%	No. 10	1	0.28%
No. 4sa	6	1.69%			
No. 4sb	4	1.13%			
No. 4sd	4	1.13%			
No. 5	11	3.11%	No. 11	2	0.56%
No. 6	14	3.95%	No. 11p	1	0.28%
			No. 11d	1	0.28%
			No. 12	3	0.85%

**Table 3:** Distribution of LNM in each station according to tumor location

Station	Upper (*n* = 6 cases)	Middle (*n* = 28 cases)	Lower (*n* = 31 cases)
No. 1	1	3	4
No. 2	2	0	0
No. 3	2	8	7
No. 4	1	7	5
No. 5	0	5	6
No. 6	0	7	7
No. 7	1	3	6
No. 8	0	1	4
No. 8a	0	1	4
No .8p	0	0	0
No. 9	0	0	1
No. 10	0	0	1
No. 11	1	1	0
No. 11p	1	0	0
No. 11d	0	1	0
No. 12	1	1	1

*LNM* lymph node metastasis

### Univariable and multivariable analysis of LNM

**Fig. 2:**
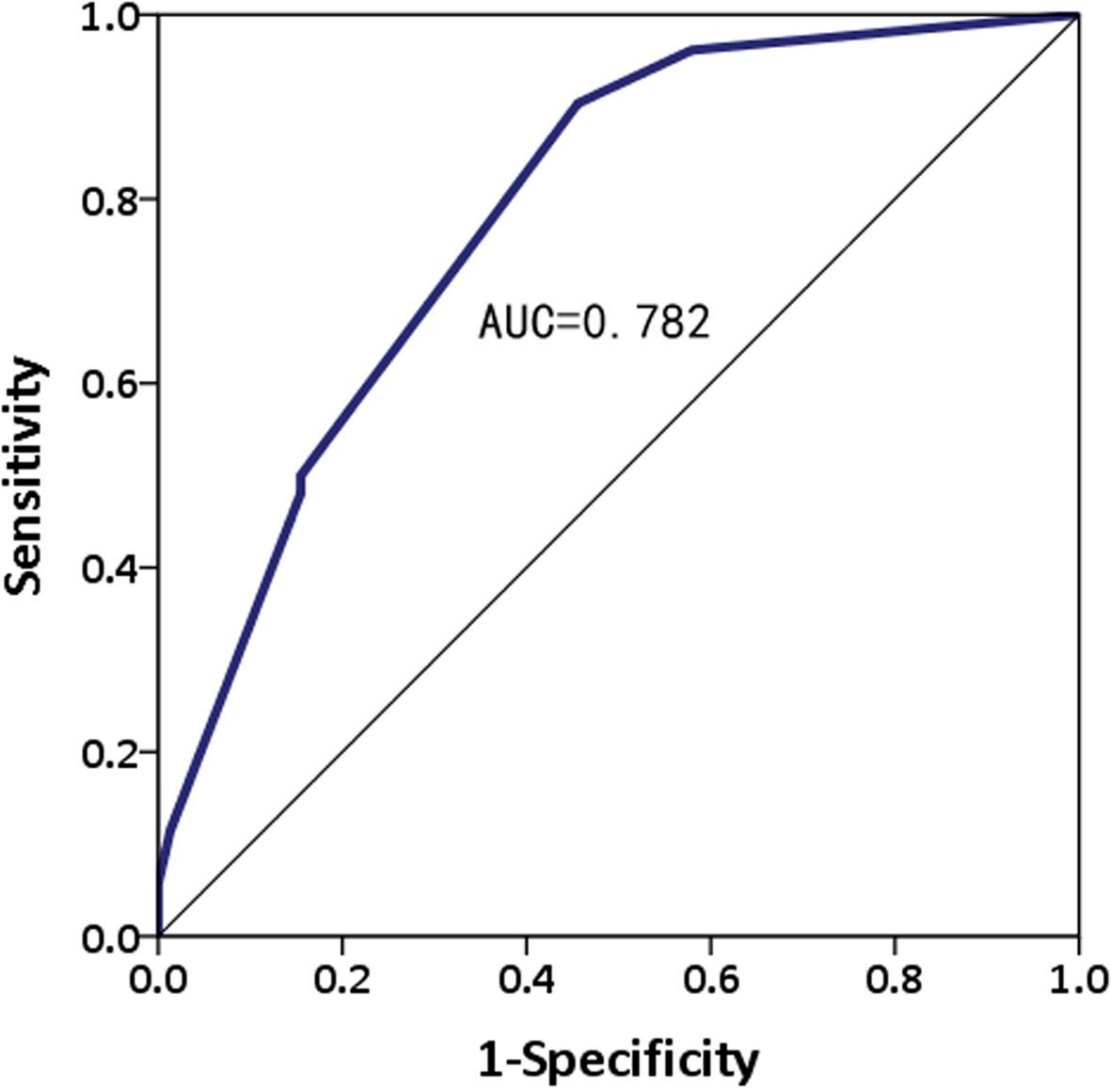
ROC curve of the multivariable model for predicting LNM. ROC, receiver operating characteristic; LNM, lymph node metastasis

**Fig. 3:**
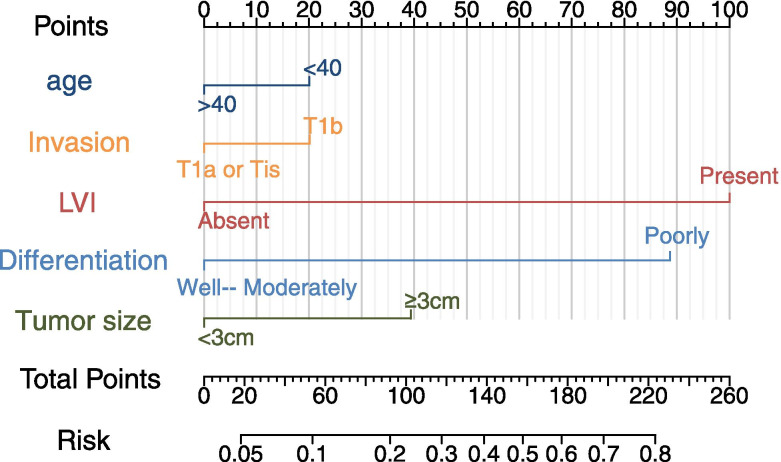
Nomogram for the prediction of lymph node metastasis

The univariable analysis showed that LNM was closely related to age (< 40 years), tumor size (> 3 cm), depth of invasion (T1b), poor differentiation*,* and LVI (all *P* < 0.05; Table 4). Multivariate analysis showed that tumor size (odds ratio [OR] = 2.948; 95% CI, 1.480–5.872; *P* = 0.002), poor differentiation (OR = 5.879; 95% CI, 2.536–13.628; *P* = 0.001), and LVI (OR = 14.569; 95% CI, 2.493–85.135; *P* = 0.001) were independent predictors for LNM (Table 4). However, age and depth of invasion were not independent predictors of LNM. The receiver operating characteristic (ROC) curve (Fig. 2) was used to validate this multivariable regression model. This model showed an area under the curve (AUC) of 0.782. Figure 3 presents a nomogram for the prediction of LNM that was constructed based on the selected variables.

**Table 4:** Univariable and multivariable analyses for LNM

Factor	Univariable analysis		Multivariable analysis	
	OR (95% CI)	*P*-value	OR (95% CI)	*P*-value
Age (years)				
< 40	1			
≥ 40	0.322 (0.139–0.748)	0.008	NA	NA
Tumor size				
< 3 cm	1			
≥ 3 cm	3.230 (1.710–6.101)	< 0.001	2.948 (1.480–5.872)	0.002
Depth of invasion				
Mucosal	1			
Submucosa	2.743 (1.583–4.755)	< 0.001	NA	NA
Ulcer				
Absent	1			
Present	0.687 (0.400–1.180)	0.173	NA	NA
Differentiation				
Well/Moderate	1			
Poorly	4.214 (2.233–7.951)	< 0.001	5.879 (2.536–13.628)	0.001
LVI				
Absent	1			
Present	8.576 (2.993–24.568)	< 0.001	14.569 (2.493–85.135)	0.001

*OR* odds ratio, *CI* confidence interval, *LVI* lymphovascular invasion, *NA* not applicable

### Correlation factors analysis of the extent of LNM

The rates of D1 and D2 station metastases in patients were 12.10% (43/354) and 6.21% (22/354), respectively (Table 5). An analysis of the clinical pathological characteristics was performed on patients with D1 or D2 station LNM. There was no significant difference between the occurrence of D2 station LNM and the age, sex, tumor size, differentiation, location, depth of tumor invasion, and LVI. The levels of CA 19–9 and CEA were significantly different between the two groups (10.113 vs. 30.125 U/mL, *P* = 0.001; 3.189 vs. 6.861 U/mL; *P* = 0.003). However, the difference in CA 125 was not significant (Table 5).

**Table 5:** Clinicopathological characteristics of the patients with D1 (*n* = 43) and D2 station metastasis (*n* = 22) set

Factor	D1 station^a^	D2 station^a^	*P*-value
Age (years)			
< 40	37	18	0.655
≥ 40	6	4	
Sex			
Male	26	12	0.647
Female	17	10	
Tumor size			
Length-diameter (cm)	2.938	2.917	0.295
Short-diameter (cm)	2.303	2.000	0.243
< 2 cm	9	2	0.163
≥ 2 cm	23	16	
< 3 cm	16	8	0.706
≥ 3 cm	16	10	
Tumor marker			
CEA (U/mL)	3.189	6.861	0.003
CA125 (U/mL)	9.702	10.568	0.165
CA199 (U/mL)	10.113	30.125	0.001
Tumor location			0.171
Upper	3	3	
Middle	22	6	
Lower	18	13	
Depth of invasion			0.322
Intra-mucosal	16	11	
Submucosa	27	11	
Differentiated			
Well-Moderately	8	6	0.421
Poorly	35	16	
LVI			
Absent	48	10	0.940
Present	9	2	

For total gastrectomy: D1: Nos. 1–7; D1+: D1 + Nos. 8a, 9, 11p; D2: D1 + Nos. 8a, 9, 11p, 11d, 12a

For distal gastrectomy: D1: Nos. 1, 3, 4sb, 4d, 5, 6, 7; D1+: D1 + Nos. 8a, 9; D2: D1 + 8a, 9, 11p, 12a

For pylorus-preserving gastrectomy: D1: Nos. 1, 3, 4sb, 4d, 6, 7; D1+: Nos. 8a, 9

For proximal gastrectomy: D1: Nos. 1, 2, 3 s, 4sa, 4sb, 7; D1+: D1 + Nos. 8a, 9, 11p

*LVI* lymphovascular invasion, *CEA* carcinoembryonic antigen, *CA125* cancer antigen 125, *CA199* cancer antigen 199

^a^According to the Japanese gastric cancer treatment guidelines 2018 (5th edition)

### Analysis of the clinicopathological characteristics of patients with skip metastasis

According to the Japanese classification of gastric carcinoma (3rd edition) [16] and the definition of skip metastasis, patients with LNM (*n* = 65) were classified into the no skip metastasis (*n* = 52) or the skip metastasis group (*n* = 13). The possibility of skip metastasis was 3.67% (13/354) in all patients. There was no significant difference between the two groups with respect to clinicopathological characteristics (Table 6).

**Table 6:** Clinicopathological characteristics of patients with LNM without (*n* = 52) and with skip metastasis (*n* = 13)

Factor	Without skip metastasis	With skip metastasis	*P*-value
Age (years)			
< 40		4	0.086
≥ 40		9	
Sex			
Male	33	5	0.102
Female	19	8	
Size			
Length-diameter (cm)	2.950	2.750	0.358
Short-diameter (cm)	2.305	1.786	0.452
< 2 cm	9	2	0.729
≥ 2 cm	30	9	
< 3 cm	20	4	0.382
≥ 3 cm	19	7	
Tumor Location			0.054
Upper	5	1	
Middle	26	2	
Lower	21	10	
Depth of invasion			
Mucosal	20	7	0.314
Submucosa	32	6	
Differentiated			
Well-Moderately	11	3	0.880
Poorly	41	10	
LVI			
Absent	45	13	0.726
Present	8	3	

*LNM* lymph node metastasis

### Univariate and multivariate analyses of prognostic factors in patients

**Fig. 4:**
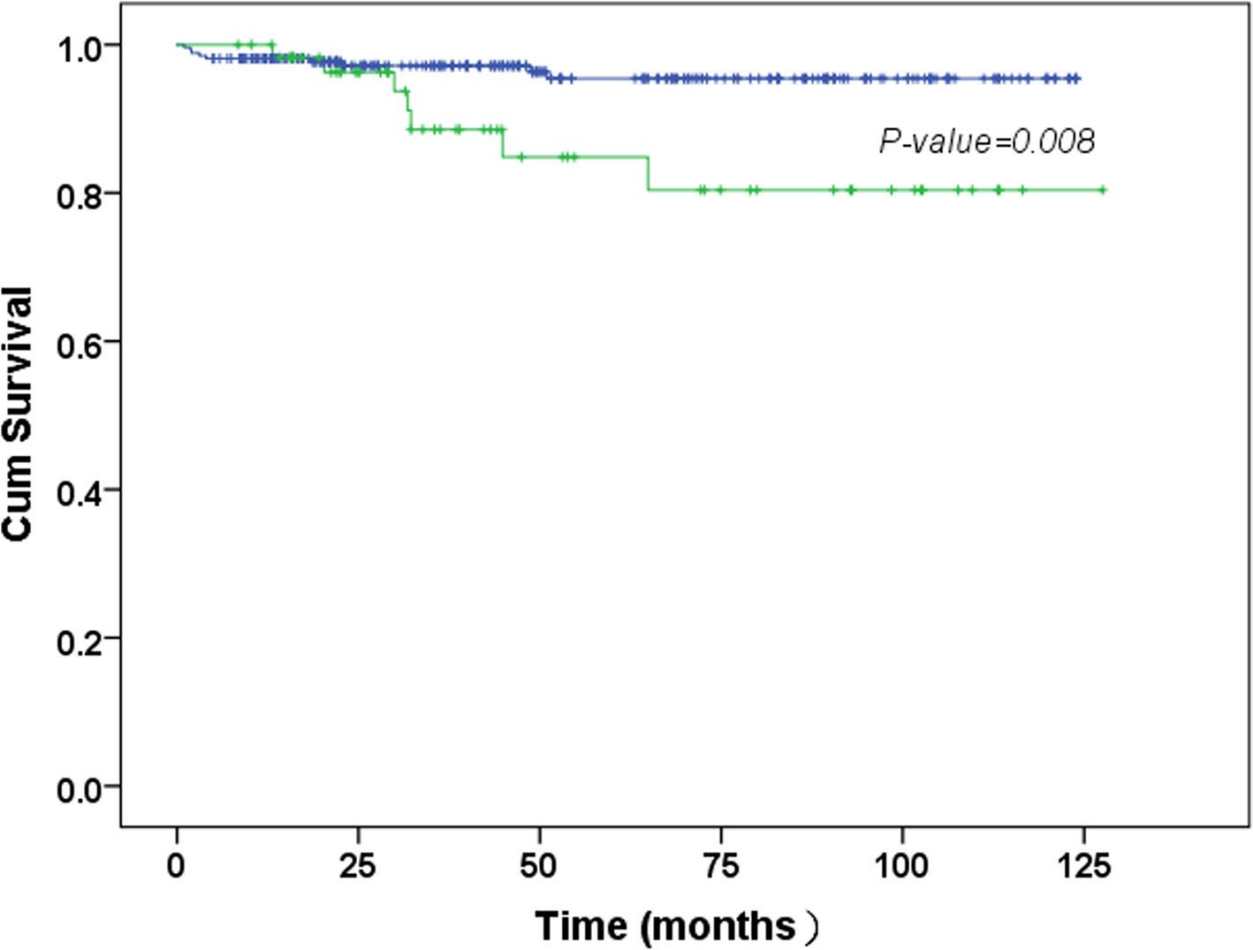
Kaplan–Meier curve of cumulative survival of patients with LNM+ (green) and LNM- (blue). LNM, lymph node metastasis; LNM-, absence of lymph node metastasis; LNM+, presence of lymph node metastasis; X-axis represents the survival time; Y-axis represents the survival rate

The 5-year overall survival rates in the LNM− and LNM+ groups were 97.19 and 83.33%, respectively (*P* = 0.021). Moreover, the disease-free survival rates in the LNM− and LNM+ groups were 96.26 and 79.17%, respectively (*P* = 0.011; Table 1). The prognostic outcome of patients who were LNM+ was worse than that of LNM- patients (*P* = 0.008) (Fig. 4). The results of the univariate and multivariate analyses for prognostic factors are presented in Table 7. Tumor size (HR, 3.473; 95% CI, 1.372–8.791; *P* = 0.009) and LNM (HR, 4.895; 95% CI, 1.588–15.095; *P* = 0.006) were independent predictive factors for poor survival outcome in all patients.

**Table 7:** Univariate and multivariate analyses of prognostic factors

Factor	Univariable analysis		Multivariable analysis	
	HR (CI 95%)	*P*-value	HR (CI 95%)	*P*-value
Age (years)				
< 40	1			
≥ 40	1.067 (0.141–8.076)	0.950	NA	NA
Tumor size				
< 2 cm	1			
≥ 2 cm	2.791 (0.937–8.317)	0.065	3.473 (1.372–8.791)	0.009
< 3 cm	1			
≥ 3 cm	1.593 (0.438–5.792)	0.480	NA	NA
Depth of invasion				
Mucosal	1			
Submucosa	1.178 (0.426–3.259)	0.753	NA	NA
Differentiation				
Well-Moderately	1			
Poorly	1.425 (0.531–3.828)	0.482	NA	NA
LVI				
Absent	1			
Present	2.419 (0.310–18.885)	0.399	NA	NA
LNM				
Absent	1			
Present	3.512 (1.307–9.438)	0.013	4.895 (1.588–15.095)	0.006

*HR* hazard ratio, *CI* confidence interval, *LVI* lymphovascular invasion, *LNM* lymph node metastasis, *NA* not applicable

### LNM rate in patients selected by the indications of ESD/EMR

The 2018 Japanese GC treatment guidelines [5] revealed that the indication for ER depends on the depth of invasion, differentiation type, diameter, and ulcerative findings. The LNM rates of these factors are demonstrated in Table 1. The data of all patients (*n* = 354) were analyzed according to the absolute and expanded indications of ESD/EMR (Table 8), and only 75 (21.18%) patients conformed to the absolute and expanded indications of ESD/EMR. The rates of LNM in absolute and expanded indications were 2/61 (3.27%) and 4/14 (28.57%), respectively. Subgroup analysis showed that the rates of LNM with respect to the absolute indication of EMR/ESD and absolute indication of the ESD 2 group were 0%. The rate of LNM with respect to the absolute indication of the ESD 1 group was 20%. For the submucosal invasive (T1b) EGC, the LNM status was analyzed with two conditions (≤2 cm, differentiated type: 7.40%; ≤2 cm, undifferentiated type: 34.375%), which was consistent with the outcome of the multivariable logistic analysis (Table 4).

**Table 8:** LNM rate of patients selected by the indications of ESD/EMR

Conditions	LNM-	LNM+	Metastasis rate
Absolute indication of EMR or ESD^a^	22	0	0%
Absolute indication of ESD 1^a^	8	2	20%
Absolute indication of ESD 2^a^	29	0	0%
Expanded indication^a^	10	4	28.57%
T1b, ≤2 cm, differentiated-type	25	2	7.40%
T1b, ≤2 cm, undifferentiated-type	21	11	34.375%

Absolute indication of ESD 1: A differentiated-type adenocarcinoma without ulcerative findings, in which the depth of invasion is clinically diagnosed as T1a and the diameter is > 2 cm

Absolute indication of ESD 2: A differentiated-type adenocarcinoma with ulcerative findings, in which the depth of invasion is clinically diagnosed as T1a and the diameter is ≤3 cm

Expanded indication: An undifferentiated-type adenocarcinoma without ulcerative findings in which the depth of invasion is clinically diagnosed as T1a and the diameter is ≤2 cm

*LNM* lymph node metastasis, *LNM-* absence of lymph node metastasis, *LNM+* presence of lymph node metastasis, *EMR* endoscopic mucosal resection, *ESD* endoscopic submucosal dissection

^a^Absolute indication of EMR or ESD: A differentiated-type adenocarcinoma without ulcerative findings (UL0), in which the depth of invasion is clinically diagnosed as T1a and the diameter is ≤2 cm

## Discussion

EGC was first defined in 1962 by the Japanese Research Society for Gastric Cancer as tumors with invasion limited to the mucosa or submucosa of the stomach, irrespective of lymph node involvement. In the 8th American Joint Committee on Cancer TNM staging system, EGC corresponds to GC with Tis, T1a (mucosa), and T1b (submucosa) stages [17]. Recently, the treatment techniques and strategies for EGC have been updated rapidly. According to the latest Japanese GC treatment guidelines (5th version) [5], EMR or ESD is considered a standard treatment for patients with EGC with absolute indications and an alternative treatment for EGC with expanded indications. With the development and prevalence of ER (ESD and EMR), the criteria for the indications of ER for EGC have continually expanded. However, there is a debate as to whether ER can be used in patients with expanded indications.

Interestingly, the 2018 Japanese GC treatment guidelines declared that the possibility of harboring LNM in the tumor with absolute indication is < 1%. However, most of the data referred to were obtained from Japanese patients [5]. It remains unclear whether the data can be extrapolated to cases from other countries. Some studies from Western countries revealed that the LNM rate of some racial/ethnic groups is almost double that of Asian patients with T1a GC [18, 19]. Here, we revealed that the incidence of LNM in patients with EGC, which confirmed the absolute or expanded indications of ESD/EMR, was obviously higher than that in the Japanese cohort [4, 20–22]. The rates of LNM in the absolute indication of the ESD 1 group (2/10, 20%) and the expanded indication group (4/14, 28.57%) were obviously > 1%. Moreover, a meta-review [4] published in 2018 indicated that the incidence rate of LNM was 2.6% (25/972) in patients who met the expanded criteria. Moreover, a Korean study [23] in 2020 reported that LNMs were found in 6.7% (18/270) of patients with undifferentiated-type EGC who underwent additional surgery after non-curative endoscopic resection. Therefore, caution should be exercised before applying ESD to patients with undifferentiated-type adenocarcinoma and those with tumors > 2 cm despite having T1a and differentiated-type adenocarcinoma without ulcerative findings. Further studies are urgently needed to find new methods to distinguish populations with high risk of LNM from EGC conforming to the indications of ER.

It is worth mentioning that the sample size of this study was small. We screened for desirable cases from 2245 patients with GC. However, only 15.77% of patients with GC were diagnosed with EGC in our center. The data were consistent with the results reported from other centers in China. The proportion of cases of EGC in China varied from 10 to 20%, compared to approximately 50% in Japan [24, 25]. Moreover, only 21.18% of patients conformed to the absolute and expanded indications of ESD/EMR in this study. The numbers of cases in the confirmed ESD 1 (*n* = 10) and expanded indication (*n* = 14) groups were too small to achieve the desired result. However, a Chinese study in 2016 also reported that the LNM rate was as high as 8.70% when the Japanese expanded criteria were used [26]. Another study reported that the rate of LNM was high (8.00–14.30%) when tumors were > 30 mm in diameter for patients with T1a stage EGC [27]. Therefore, our data suggested that ESD/EMR treatment of EGC should be considered carefully in different racial populations [19], and more data are needed to draw a firm conclusion about the expanded indication for ESD.

The Japanese Gastric Cancer Association guidelines suggested a gastrectomy procedure with D1/D1+ lymph node dissection as the standard surgical procedure for cases, in which the depth of invasion is clinically diagnosed as T1b without LNM and T1a without LNM, which do not allow the performance of EMR and ESD. However, our data showed that the rate of D2 station LNM was 6.21%. For these cases, the D1 or D1+ dissection is not sufficient. Furthermore, skip LNM is another factor influencing the determination of the extent of lymph node dissection.

Skip metastasis in GC refers to the presence of extraperigastric LNM without perigastric lymph node involvement [10]. There have been few studies on the phenomenon of jump metastasis and its related mechanism in patients with GC, especially in those with EGC [9–13, 28]. The incidence of skip metastasis in patients with LNM in EGC has been reported to range from 2.7 to 21.6% [10, 28, 29]. In this study, the incidence of skip metastasis in patients with LNM was 3.67% (13/354), which is consistent with prior research results. Liu et al. [9] revealed that tumor size was the only clinicopathologic factor that could predict lymph node skip metastasis in patients with N1 stage cancer (the number of metastatic lymph nodes among the regional lymph nodes is 1–2) undergoing radical surgery. However, no significant related clinical characteristics were found for skip metastasis in our study. Considering the relatively high incidences of D2 LNM and skip LNM in EGC, it is not suitable for these patients to receive D1 or D1+ dissection. Therefore, the identification of these high-risk portions from EGC is urgently needed so that the patients can undergo radical lymphadenectomy.

Regarding the extent of gastric resection, the Japanese GC treatment guidelines have revealed that the standard surgical procedure for cN+ or T2-T4a tumor is total or distal gastrectomy. For cT1N0 tumors, PPG and proximal gastrectomy can be considered depending on the tumor position. PPG is a less-invasive function-preserving procedure that has been applied for the cT1N0M0 middle-third EGC with a distal tumor border at least 4 cm proximal to the pylorus according to the Japanese GC treatment guidelines [5]. The survival and recovery benefits of PPG have already been reported in several retrospective studies [30–32]. However, the performance of PPG remains controversial. One of the reasons is that the dissection of the No. 5 lymph node may be incomplete when performing PPG because the pyloric branches of the vagus nerve are kept to reduce the postoperative gastric stasis complications. A previous study reported that the metastasis rate of No. 5 lymph node in middle-third EGC was only 0.5% [33]. Kong et al. [34] reported that the metastasis rates of the No. 5 lymph node in middle-third EGC with a distal tumor border at least 6 cm proximal to the pylorus were 0% in T1a stage and 0.9% in T1b stage EGC. However, the metastasis rate of the No. 5 lymph node was 3.03% (5/165) for the middle-third EGC in this study (Table 2 and Table 3), which was similar to the result of Seung et al. [35], who reported that the metastasis rate to the No. 5 lymph nodes was 4.2% (52/1245). Seung et al. [35] also pointed out that the presence or absence of metastasis in the No. 5 and No. 6 lymph nodes should be carefully evaluated preoperatively using endoscopic ultrasonography and CT. Therefore, caution should be exercised before performing PPG for EGC given that the risk of No. 5 LNM is high according to our data. Further prospective studies using large case series are necessary to confirm this conclusion. It is worth mentioning that PPG should be performed for GC located in the middle third of the stomach and at least 4.0 cm away from the pylorus according to the guidelines. However, information about the distance from the tumor to the pylorus was unavailable in our database. This limitation weakened our conclusion.

LNM is one of the most important factors influencing the prognosis of EGC. The risk of LNM is a major concern when choosing the optimal treatment for EGC. According to previous studies, the incidence of LNM in patients with EGC, regardless of T1a or T1b diagnosis, was 15–24% [36, 37]. Recently, Chen et al. reported that the LNM rate was 16.7% in EGC (8.7% in T1a, 24.6% in T1b) in their retrospective study that enrolled 1033 patients with EGC [38]. In agreement with results of previously reported studies, our data showed that the incidence rates of LNM were 12.38% in T1a stage, 27.94% in T1b stage, and 18.36% in whole EGC. Classifying the low and high risks of LNM in patients with EGC is important in EGC studies.

A number of studies have identified the risk factors associated with LNM in EGC [28, 39–43]. They also revealed that LNM in EGC is related to differentiation, tumor size, depth of invasion, and LVI, which is consistent with the results of this study (Table 4).

The AUC of the ROC curve (Fig. 2), which validated this multivariable regression model, was 0.783 in this study. In other studies on the prediction of LNM with clinicopathological characteristics, the AUCs of the ROC curves were approximately 0.69–0.86 [40, 44, 45]. Similarly, the 2018 Japanese GC treatment guidelines [5] predicted the risk of LNM in EGC according to the clinicopathological characteristics including histological types (ulcerative findings), and tumor sizes < 2 cm (non-ulcerative) and < 3 cm (ulcerative). However, the prediction of LNM in EGC by these factors is still not ideal given that the incidence risk of LNM was high in the population of patients with EGC with absolute or expanded indication, as shown in this study. The prediction of LNM in EGC based only on the current routine detection items and pathological examination may not be reliable. Hence, the discovery of more factors that could more accurately predict LNM remains a research interest in the field of EGC. Finally, this study also analyzed the prognosis of patients with EGC and revealed that those with LNM had a worse prognosis (Fig. 4).

## Conclusions

In summary, the risks of LNM were high in patients with EGC with undifferentiated-type adenocarcinoma and in those with a > 2-cm tumor and expanded indications of ER. In addition, PPG remains controversial due to the high metastasis rate of the No. 5 lymph node in patients with middle-third tumor. Hence, physicians should be cautious when choosing a minimally invasive treatment (e.g., EMR, ESD, or PPG) that could carry a risk if the dissection of metastatic lymph nodes is neglected.

## Data Availability

The datasets used and/or analyzed in the current study are available from the corresponding author on reasonable request.

## Abbreviations

EGC: Early gastric cancer
GC: Gastric cancer
LNM: Lymph node metastasis
LVI: Lymphovascular invasion
EMR: Endoscopic mucosal resection
ESD: Endoscopic submucosal dissection
PPG: Pylorus-preserving gastrectomy
CA: Cancer antigen
CEA: Carcinoembryonic antigen
HR: Hazard ratio
CI: Confidence interval
OR: Odds ratio
ER: Endoscopic resection
RR: Risk ratio
ROC: Receiver operating characteristic
AUC: Area under the curve
CT: Computed tomography
